# The Effect of the Composition of a Concrete Mixture on Its Volume Changes

**DOI:** 10.3390/ma14040828

**Published:** 2021-02-09

**Authors:** Martin Ťažký, Lenka Bodnárová, Lucia Ťažká, Rudolf Hela, Milan Meruňka, Petr Hlaváček

**Affiliations:** 1 Faculty of Civil Engineering, Institute of Technology of Buildings Materials and Components, Brno University of Technology, Veveri 331/95, 602 00 Brno, Czech Republic; tazky.m@fce.vutbr.cz (M.Ť.); bodnarova.l@fce.vutbr.cz (L.B.); hela.r@fce.vutbr.cz (R.H.); merunka.m@fce.vutbr.cz (M.M.); 2 Department of Material Disintegration, Institute of Geonics of the CAS, Studentska 1768, 708 00 Ostrava, Czech Republic; petr.hlavacek@ugn.cas.cz

**Keywords:** concrete, volume changes, shrinkage, crushed and mined aggregate, finely ground limestone, finely ground granulated blast furnace slag, coal high-temperature fly ash

## Abstract

The presented research aims to clarify the specific effect of the individual components of concrete with Portland cement CEM I 42.5 R on the volume changes of concrete. The effect of the filler component was evaluated from the point of view of the composition and type of aggregate (crushed versus mined) and from the point of view of the mineralogical composition of the aggregate. Concrete formulas with a maximum aggregate grain size of 16 and 22 mm were assessed. The effect of the binder component on the shrinkage of the concrete was monitored on the concrete mixtures produced using the same aggregate and maintaining the same strength class of concrete, C 45/55. The effect of the addition of finely ground limestone, finely ground granulated blast furnace slag and coal high-temperature fly ash was monitored. It was found that the maximum aggregate grain and the type of grading curve do not have a significant effect on the volume changes of concrete. Concretes with mined aggregates showed lower shrinkage than concretes with crushed aggregates. The most significant is the effect of the type of aggregate on the volume changes in the first 24 h. Mineral additives have a positive effect on the elimination of the volume changes of concrete, while the addition of high-temperature fly ash proved to be the most suitable.

## 1. Introduction

The widespread usage of concrete around the world is mainly due to its excellent formability in the fresh state and its good mechanical parameters and durability in its hardened state. However, the resulting mechanical parameters and durability of the concrete composite are affected by its microstructure without signs of micro-defects. One of the most common micro-defects of cement composite is the formation of micro-cracks due to excessive volume changes and the production of hydrating heat. Thus, it can be said that the good mechanical resistance and the resulting durability of the concrete composite depends on its hydration processes and the volume changes that have taken place during this hydration [1].The resulting micro-cracks due to excessive volume changes become a place for the penetration of aggressive media from the surrounding environment into the micro-structure of concrete, which has a negative impact on the durability of the composite [2]. In addition, micro-cracks occurring on the surface of the composite adversely affect its applicability for architectural purposes. A significant negative impact of micro-cracks is the reduction of the resistance of concrete to mechanical abrasion and to abrasion caused by flowing liquids, while in micro-cracks, the further development of discontinuities occurs due to contact with abrasive particles and flowing media. Therefore, one of the important requirements for concretes for the construction of dams, drainage tunnels, sewers, canals and Barbour constructions, etc., is to achieve a low-shrinkage value to prevent cracking in these massive and highly stressed structures [1,3].

The volume changes of the concrete composite can be divided into several stages, which follow each other or overlap [4,5]. In general, these volume changes can be divided into practically uncontrollable and controllable. The first mentioned include chemical and autogenous volume changes, which are the basic processes accompanying the hydration processes of Portland cement [6,7,8]. This type of shrinkage is, therefore, directly dependent on the type of cement and its parameters, such as its chemical and mineralogical composition and its fineness of grinding [9,10]. Expert publications based on worldwide research attribute to this type of shrinkage up to 20% of the total volume changes of the concrete composite, although its size can hardly be influenced by the composition of the concrete mixture alone, if we omit the possible partial replacement of Portland cement with an active additive [11,12,13].

The highest part of the total volume changes of the concrete composite, according to the performed studies, falls on shrinkage due to drying [14,15,16]. This type of shrinkage is already directly dependent on the treatment of the composite after its production, the shape of the structure and exposure to ambient conditions [14,15,16]. However, the composition of the concrete mixture itself can also have a direct impact on this process of volume changes. Published results often look for connections between the consistency of a fresh concrete mixture, more precisely the water–cement ratio and volume changes due to drying. In general, the conclusion is often published that with an increasing water–cement ratio, the volume changes of the composite increase mainly by drying [17,18,19,20]. However, other studies point to the danger of excessive reduction of the water–cement ratio due to strong superplasticizers, which may mean danger in terms of long-term volume changes of the composite [21,22].

Although a number of studies have dealt with the impact of the type of used aggregate and its grading curve on the volume changes of the composite, it is not possible to clearly determine the intersection of the obtained results. Some research provides a picture of the suitability of crushed aggregates, which can have a positive effect on volume changes in the composite. Others have favored the use of quality mined aggregates, which can achieve a comparable degree of consistency through a lower dose of water and a lower amount of fine fractions. This theory generally applies, especially to the higher degrees of the consistency of fresh concrete, which is a trend in ready-mixed concrete today, and it relies on the theory of a lower specific surface of mined aggregate grains with a spherical grain shape compared to crushed aggregates. This fact results in a lower required dose of water for wetting the surface of aggregate grains. This statement is supported by the research of Polat et al. [23], where 4 specific grain shapes of aggregates, spherical, flat, elongated and mix type aggregates, in concrete mixture with uniform water–cement ratio (w/c) = 0.3 were investigated. The research showed that the best workability of the fresh concrete by the slump test was for aggregates with a spherical grain shape (same as mined aggregate) of 150 mm and, conversely, the worst workability with elongated aggregates (crushed), namely 110 mm [23]. As a result, lower water–cement ratio values can generally be applied, which has a direct impact on the volume changes of the composite [24,25,26].

The presented research aims to clarify the specific effect of the individual components of concrete with Portland cement CEM I 42.5 R on the volume changes of concrete. The article is divided into two parts. The first part solves the influence of different types of aggregates on the shrinkage of concrete. The effect of the filler component was evaluated from the point of view of the composition and type of aggregate (crushed versus mined) and from the point of view of the mineralogical composition of the aggregate. Concretes with different mineral aggregate type—psammite, quartz, amphibole and granodiorite—were compared. Concrete formulas with a maximum aggregate grain size of 16 and 22 mm were assessed. When crushed and mined, aggregates were used, and the concretes were monitored, which showed the same grading curve of the aggregate mixture. The second part of the study is directly related to the first part, and it is devoted to the influence of binders on the shrinkage of concrete. The effect of the binder component on the shrinkage of the concrete was monitored on the concrete mixtures produced using the same aggregate and maintaining the same strength class of concrete, C 45/55. The effect of the addition of finely ground limestone, finely ground granulated blast furnace slag and coal high-temperature fly ash was monitored.

The innovativeness and usefulness of this study is seen in the complexity of the solution of this experiment, which in the second part deals with the influence of aggregates and subsequently the influence of mineral admixtures on the shrinkage of concrete. Another significant benefit or novelty is focused on the possibility of measuring volume changes immediately from the fresh state. The results presented in this experiment demonstrate that most of the volume changes take place in the fresh state, which is in conflict with some standardization methods, which consider the beginning of measurement to measurement after gaining handling strength from 1 day of age. If the initial shrinkage is not captured, the results of volume changes may differ significantly, which may affect the final similarity of concrete structures. We consider this fact to be especially important from the point of view of concreting, for example, large expansion units, where the architect’s requirement for minimal volume changes is increasingly being taken into account today.

## 2. Materials and Methods

### 2.1. The Properties of the Aggregate

Aggregate forms a filler component in concrete and occupies the largest part of its volume, namely 70% to 80% [27]. Although aggregate can be classified as an inert material from the point of view of ongoing hydration and chemical processes, it can have a fundamental effect on the course of volume changes due to its largest share in the concrete composite. From the point of view of the effect of the aggregate on the volume changes of concrete, the effect of the maximum grain size and the type of grading curve of the aggregate mixture is discussed in worldwide research [28,29]. The majority of produced concrete mixtures are composed of at least two fractions of aggregate, where one fraction is fine aggregate, i.e., up to a maximum grain size of 4 mm, and the other fraction is coarse aggregate, with a larger aggregate grain size. The most commonly used fractions of coarse aggregates in Central Europe include fractions of 4–8, 8–16, 11–22 or 16–22 mm. These fractions can be mixed as needed with each other, or some of them can be omitted when creating the resulting grading curve; thus, continuous, or discontinuous grain curves are formed [28,29].

In the performed experiment, the effect of the type of grading curve (continuous versus discontinuous) and the effect of the maximum aggregate grain, specifically D_max_ 16 mm and D_max_ 22 mm, were assessed [30].

Furthermore, the effect of the used type of aggregate was evaluated in terms of its production (mined versus crushed) in relation to its mineralogy and physical-mechanical parameters.

For the evidence and practical application of this experiment, a total of four formulas with different mined aggregates and four formulas with different crushed aggregates were produced. The selection of individual aggregates was based on the portfolio of the most common types of aggregates based on their origin and occurrence in the Czech Republic. Within the mined aggregates, excavated sedimentary psammitic rock (locality Zabcice, Czech Republic) and sedimentary rock extracted from water, mostly of the quartz type with an additive of quartzite and marl (locality Naklo, Czech Republic), were selected. Furthermore, natural crushed aggregate of the deep igneous amphibolite rock type (clastic clays + quartz feldspars, locality Zelesice, Czech Republic) and metamorphic rock of the granodiorite type (locality Olbramovice, Czech Republic) were used. All aggregates meet the requirements of the standards for aggregates for concrete production according to EN 12620 + A1 and EN 13242 for aggregates for civil engineering and infrastructure [30,31]. The parameters of used aggregates are shown in Table 1 and Table 2.

**Figure 1:**
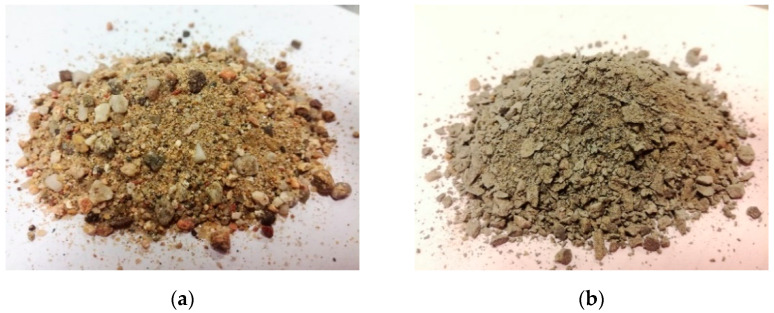
Photographs of grain shape of used small aggregates: (**a**) psammitic mined rock (Zabcice, CZ) and (**b**) crushed amphibolite rock (Zelešice, CZ).

**Figure 2:**
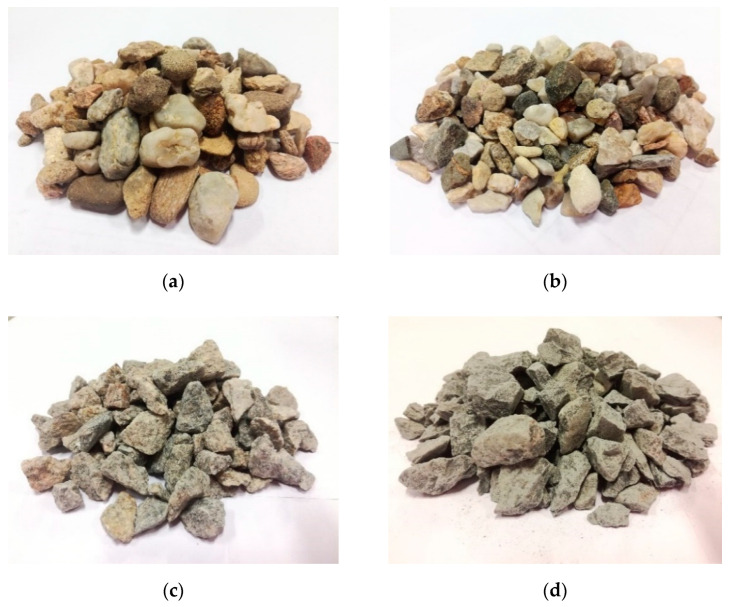
Photographs of grain shape of used coarse aggregates: (**a**) psammitic mined rock (Zabcice, CZ), (**b**) quartz mined rock (Naklo, CZ), (**c**) crushed granodiorite rock (Olbramovice, CZ) and (**d**) crushed amphibolite rock (Zelesice, CZ).

The individual types of aggregates and their shapes are shown in Figure 1 and Figure 2. The exact physical parameters of the aggregates used were verified by several tests. The basic tests performed included the determination of the density of the aggregates and the water absorption according to EN 1097-6 [32], a sieve analysis according to EN 933-1 [33] and for coarse aggregates, by the determination of the grain shape—shape index according to EN 933-4 [34]. The results are shown in Table 1 and Table 2. From the point of view of the volume changes of concrete, the shape of its grains and its water absorption can be considered as key properties and possibly also the cumulative specific surface of grains, which is given not only by the representation of the grains of individual sizes (grain category) but also, to some extent, by their shape index.

For the other parts of the experiment, the following markings of the individual types of aggregates are introduced:

- Psammitic mined rock—PM
- Quartz mined rock—Q
- Crushed amphibolite rock—AM
- Crushed granodiorite rock—GR

From the photographs of the grain shape and the determination of the grain shape by the shape index test, it is clear that there is a significant difference between mined and crushed aggregates in general. From this point of view, it can be concluded that the used crushed aggregates reach a significantly higher specific surface than the mined aggregates, and higher doses of mixing water will be needed to moisten. It can be assumed that this fact will be most evident in concrete mixtures where crushed fine aggregate of the amphibolite type will be used. Although the usability of this fraction of crushed aggregate for the production of standard ready-mixed concretes is practically negligible, the possibility of its higher use and the impact of its use on the volume changes of concrete will be verified within the performed experiment.

The higher specific surface of the aggregate grain, which can be easily quantified for conventional natural aggregates by a shape index value (especially for crushed aggregates), creates a larger area for incorporating the aggregate grain into cement stone [35]. However, some studies state that due to the higher surface of the aggregate grains, a higher water–cement ratio is needed to maintain the required degree of consistency of the concrete mixture, which can have a negative effect on the transit zone of the aggregate grains. Cement putty in the area of the transit zone always reaches a higher water–cement ratio due to water adhering to the surface of the aggregate grain, which can lead to higher values of volume changes of the composite, as described in the introduction [36].

Furthermore, based on the results given in Table 1 and Table 2, it is possible to expect the need to increase the water–cement ratio for mined aggregates due to their increased value of water absorption compared to the crushed aggregates used. Aggregates of psammitic origin from the Zabcice locality show the highest water absorption of all analyzed and used aggregates for the experiment. As part of the evaluation of the results of the volume changes of the produced concretes using the analyzed types of aggregates, the context between shape index, water absorption and the resulting value of volume changes of the composite will be sought.

To determine the effect of aggregate on the volume changes of concrete, a total of eight formulas were designed in accordance with the EN 206+A1 standard [37]. All concrete mixtures were designed for strength class C 45/55. This higher strength class was chosen due to the use of higher doses of a binder component, where the higher values of the volume changes can be expected for these mixtures. Thanks to this, the obtained results will be more conclusive.

In the experiment, four concrete mixtures with a continuous grading curve and a maximum grain size of 16 mm and another four concrete mixtures with a discontinuous grading curve and a maximum grain size of 22 mm were designed, omitting the 4–8 mm aggregate fraction. The individual formulas differ from each other in the type of aggregate used.

The following mixtures of aggregates were used in the performed experimental measurements:

- M-PM with D_max_ 16 mm—fraction 0–4 (PM), 4–8 (PM) and 8–16 mm (PM)
- M-PM with D_max_ 22 mm—fraction 0–4 (PM), 8–16 (PM) and 16–22 mm (PM)
- M-PMQ with D_max_ 16 mm—fraction 0–4 (PM), 4–8 (Q) and 8–16 mm (Q)
- M-PMQ with D_max_ 22 mm—fraction 0–4 (PM), 8–16 (Q) and 11–22 mm (Q)
- C-PMAM with D_max_ 16 mm—fraction 0–4 (PM), 0–4 (AM), 4–8 (AM) and 8–16 mm (AM)
- C-PMAM with D_max_ 22 mm—fraction 0–4 (PM), 0–4 (AM), 8–16 (AM) and 11–22 mm (AM)
- C-PMGR with D_max_ 16 mm—fraction 0–4 (PM), 4–8 (GR) and 8–16 mm (GR)
- C-PMGR with D_max_ 22 mm—fraction 0–4 (PM), 8–16 (GR) and 11–22 mm (GR)

The letter M in front of the mixture indicates the mixture with mined aggregate, and the letter C indicates the mixture with crushed aggregate.

**Figure 3:**
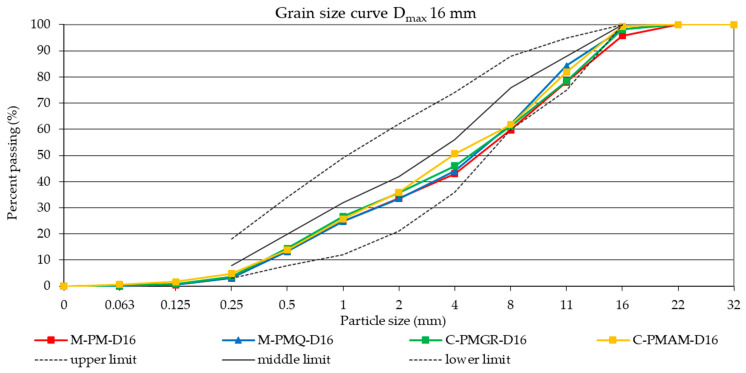
The resulting grading curves of aggregate mixtures with a continuous grading curve and D_max_ 16 mm.

**Figure 4:**
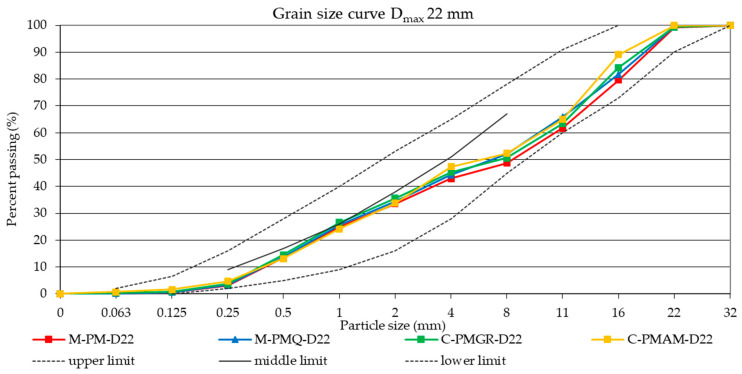
The resulting grading curves of the mixtures of aggregates with a discontinuous grading curve and D_max_ 22 mm.

For all mixtures of aggregates with a continuous grading curve and D_max_ 16 and with a discontinuous grading curve and D_max_ 22, the effort was to achieve the same grading curve. These grading curves are shown in Figure 3 and Figure 4. The design of the same grading curve for the individual formulas with a continuous or discontinuous grading curve was chosen because only a different parameter from the perspective of the composite filler was the type of aggregate used, and its effect could thus be precisely determined.

All the concrete mixtures were produced using a constant amount of Portland cement CEM I 42.5 R from the company Heidelberg Cement, cement plant Mokra, Czech Republic. The total calculation of the composition of the concrete mixture is based on the equation of absolute volumes, and the amount of water and superplasticizer is chosen with regard to achieving the workability of a fresh concrete mixture using a slump cone test for class S4 according to EN 12350-2 [38]. A superplasticizer based on polycarboxylate ether from MC Bauchemie (Bottrop, Germany) was used for the experiment. The properties of the used cement and superplasticizer are contained in Table 3. An overview of the composition of the individual mixtures is given in Table 4 and Table 5.

### 2.2. The Properties of Mineral Additives

In following part of the experiment, connections between commonly used mineral additives in the middle Europe and volume changes of the composite will be sought. Today’s concrete technology is strongly focused on the use of additives directly in the concrete plants or as part of mixed cements. According to the EN 206 + A1 standard, additives can be divided into two types: an inert additive of type I and an additive with pozzolanic or latent hydraulic capability type II. The use of additives to produce concrete is not only given by the economic aspect, most additives are also used to positively influence selected properties of fresh or hardened concrete. The experiment will study the effect of the most commonly used inert and active additives on the volume changes of concrete. It is known to use certain types of additives, such as high-temperature fly ash, to dissolve the development of hydration temperatures in massive structures. For these types of additives, their positive impact on the resulting volume changes of the composite can also be expected. This theory is based mainly on a slower course of hydration processes and a possible reduction in the dose of the main binder component, Portland cement, while maintaining comparable mechanical parameters of concrete [39,40].

The following mineral additives originating from the production of producers available in the Czech Republic were selected for the experiment:

- Very finely ground limestone—locality Mokra
- Very finely ground granulated blast furnace slag—locality Detmarovice
- Coal high-temperature fly ash—locality Detmarovice

**Figure 5:**
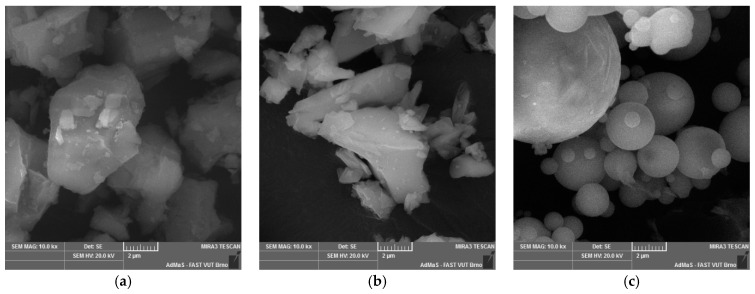
Photographs of the grain morphology of the analyzed mineral additives: (**a**) very finely ground limestone (Mokra, CZ), (**b**) ground blast furnace granulated slag (Detmarovice, CZ), (**c**) high-temperature fly ash (Detmarovice, CZ)—magnification 10,000 times.

The basic mechanical and physical parameters, such as the efficiency index, the specific surface and loss on ignition, were determined for the mineral additives used, and the percentage of basic oxides was determined by XRF (X-ray fluorescence, device Axios PANalytical, Malvern, UK). All these parameters are shown in Table 6 and Table 7. The mineral additives used meet the relevant normative parameters for use as additives in concrete according to EN 206+A1. To illustrate, all the mineral additives used were further observed under a scanning electron microscope, and the following photographs in Figure 5 show their different grain morphology.

The determined efficiency index was performed by replacing 25% by weight of the Portland cement with the given additive. The results show significant activity of the blast furnace slag, but they also point to the partial activity of finely ground limestone, which from the point of view of legislation is considered an inert additive of type I. These results for very finely ground limestone agree with some published studies, which attribute to it a hidden activity in the sense of the formation of crystallization seeds during the hydration of the cement [41,42].

As part of the further verification of the effect of the used aggregate type (mined versus crushed), all the formulas designed for this experiment were always made with a discontinuous grading curve and Dmax 22 mm with mined quartz aggregate (locality Naklo, Czech Republic) and crushed granodiorite aggregate (locality Olbramovice, Czech Republic) shown in Figure 4. Following the previous determination, the same grading curves of the aggregate mixtures and the same composition of the comparative concretes were maintained. Thanks to the production of the concrete mixtures of the same consistency and the same compressive strength after 28 days, it is possible to assess only the effect of a given mineral additive on the subsequent volume changes of concrete.

Portland cement CEM I 42.5 R from the manufacturer Heidelberg Cement in Mokra and superplasticizing additives from the company MC Bauchemie PowerFlow 2695 (Bottrop, Germany) were also used in this part of the experiment. The following mixtures of aggregates were used in this experiment:

- M-PMPQ with D_max_ 22 mm—fraction 0–4 (PM), 8–16 (Q) and 11–22 mm (Q)
- C-PMGR with D_max_ 22 mm—fraction 0–4 (PM), 8–16 (GR) and 11–22 mm (GR).

Table 8 contains the composition of all the concrete mixtures being compared.

### 2.3. Testing Methods

For the produced concretes in their fresh state, their density was determined according to EN 12350-6 [43] and the degree of consistency by the slump cone method according to EN 12350-2 [38] within five minutes of production.

**Figure 6:**
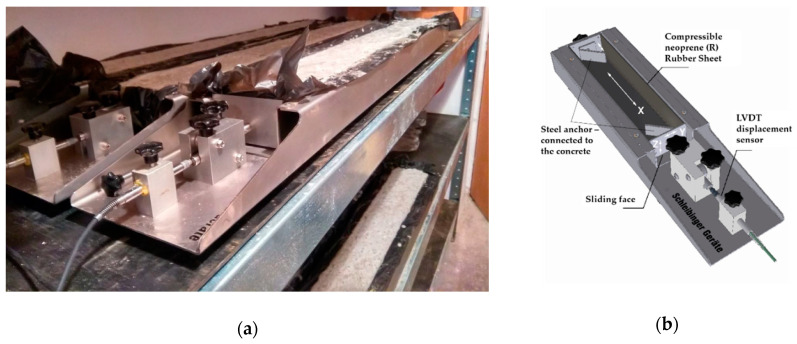
Device for measuring the volume changes using a shrinkage drain from Schleibinger Geräte: (**a**) material measuring, (**b**) scheme of equipment [50].

The main part of both experiments was to determine the volume changes of the concretes produced. This determination was carried out immediately from the fresh state to the age of 28 days in case of different types of aggregates and the age of 60 days in case of mineral admixtures. The measurement was determined by using special molds with a U-shaped cross-section and dimensions of 100 mm × 60 mm × 1000 mm (width × height × length). These molds from Schleibinger Geräte’s company (Buchbach, Germany)—shrinkage drain—have a sliding face, which is firmly connected to the concrete mixture through a concreted steel anchor, and the shrinkage of the concrete is thus manifested by the forced displacement of this face. A strain gauge sensor is attached to the sliding face, and the current value of the volume change of the mixture is automatically recorded every five minutes for a specified time. The filled test molds were placed in a room with a constant relative humidity of 50% and a constant ambient temperature of 20 °C throughout the measurement. The device is shown in Figure 6. This test procedure is in accordance with ÖNORM B 3329 [44]. Three test specimens were made from each test mixture, and the result is their average value.

- Density of hardened concrete after 7 and 28 days according to EN 12390-7 [45]
- Compressive strength (device ratioTEC RT3000-1 ST servo, Langenenslingen, Germany) after 7 and 28 days according to EN 12390-3 [46]
- Flexural strength (device ratioTEC RT 200-1 D servo, Langenenslingen, Germany) after 7 and 28 days according to EN 12390-5 [47]
- Water absorption after 7 and 28 days according to CSN 73 1316 [48]
- Water tightness HV8 after 7 and 28 days according to TKP RVC CR [49]

The same test procedures showed the difference in determining the properties of hardened concrete even after 90 days for mixtures with different mineral additives. Samples for these tests were stored in an aqueous medium at a constant temperature of 20 ± 2 °C throughout the maturation period. From each test mixture, three test specimens were made for each test, and the result is their average value.

The watertightness of concrete to grade HV8 was determined according to the regulation of the Waterways Directorate of the Czech Republic [49]. This test consists of the ability of concrete to withstand a water pressure of 400 kPa for the first 24 h and then 800 kPa for a further 48 h. After the test, the test specimen is broken perpendicular to the action of the water pressure, and the maximum depth of leakage is determined.

## 3. Results and Discussion of Results

### 3.1. The Effect of the Aggregate on the Volume Changes of Concrete

All produced concrete mixtures reached the degree of consistency S4 through the slump cone test, more specifically, a slump of about 190 mm. To achieve the same degree of consistency for all produced concretes, it was necessary to make small corrections to the water–cement ratio and the dose of the superplasticizer, as can be seen in the Table 4 and Table 5 with the composition of the individual formulas. As expected, a higher dose of water and superplasticizer was needed for the crushed aggregates. More specifically, it was an increase in the water dose by up to 7 kg/m^3^ of concrete. This increase was necessary for the mixture containing (excluding the mined fine aggregate) crushed fine aggregate, and it can be expected that this increased dose was necessary due to the wetting of the surface of all aggregate grains. These results are in accordance with the general determinations regarding the specific surface of the aggregate described above. Also, in the following evaluation of the volume changes of the concrete composites, the connections between the parameters of the aggregate, such as grain shape, and the absorptivity and achieved volume changes will be further sought.

The Table 9 show the same workability was obtained in all mixtures. In mixtures with mined aggregates, such as PM and PMQ, less water content was used than in other mixtures. This result is in correlation with the statement made in the introduction [23].

**Figure 7:**
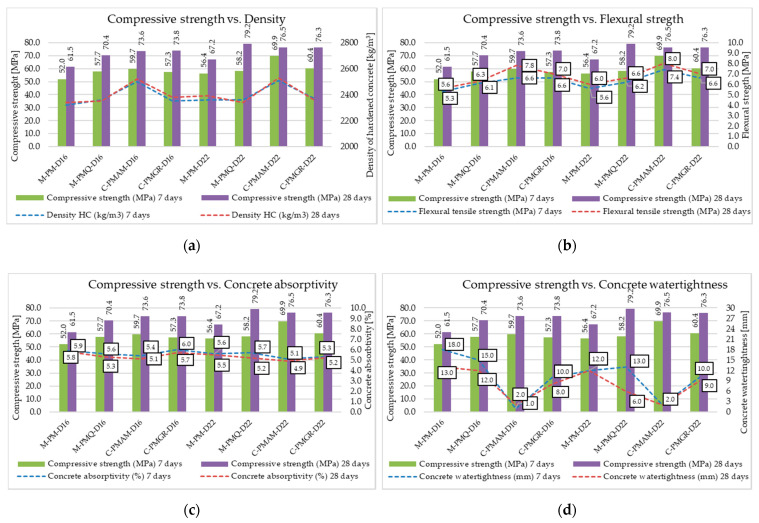
Mechanical and physical parameters of tested concretes’ compressive strength versus (**a**) density of hardened concrete, (**b**) flexural tensile strength, (**c**) concrete absorptivity and (**d**) concrete water tightness.

According to the achieved results of the compressive strength of concrete, it can be said that the required strength class was achieved for all the formulas. However, the achieved results showed in Figure 7, clearly point to the effect of the type of aggregate, especially on the strength parameters of concrete. According to the achieved results, the influence of the bulk density of the type of aggregate used on the resulting bulk density of the concrete is obvious. Aggregates with the highest bulk density (Zelesice—amphibolite type) increased the bulk density of concrete to a value of around 2500 kg/m^3^. The results show a significant difference between the compressive strength of concrete made from psammitic fluvial rock of the mined type and the remaining aggregates used. Based on these results, it can be concluded that it was the lowest quality aggregate of all the aggregates used in terms of its mechanical properties. On the contrary, no significant difference in the compressive strength of concrete was observed when using high-quality quartz-type mined aggregate compared to high-quality crushed aggregates used in this experiment. The results further showed a slight increase in the mechanical parameters of all produced concretes using a discontinuous grading curve and a larger maximum aggregate grain size, namely D_max_ 22 mm. From a practical point of view, the formation of a discontinuous grading curve omitting the 4–8 mm fraction appears to be economically advantageous and, according to the results, is suitable for the production of concretes of higher strength classes.

Furthermore, a significant difference in the flexural strength of concrete was observed between formulas made with mined aggregates and formulas made with crushed aggregates. It is obvious that the assumed larger specific surface of coarse aggregates, according to the achieved results, may seem more suitable for achieving higher values of flexural properties. These results correlate with the conclusion about a better incorporation of crushed aggregate into the matrix of cement stone, which can have a positive effect on the flexural strength of concrete. However, these obtained results probably also correspond to the mechanical parameters of the aggregates used, whereby metamorphic rocks of granodiorite type and igneous rocks of amphibolite type generally achieve significantly higher mechanical parameters than mined rocks. Therefore, it is not possible to unequivocally confirm or disprove these conclusions about increasing the bending parameters of concrete by better incorporating the grains of crushed aggregates into the cement matrix. The effect of the maximum aggregate grain size and the continuous or discontinuous aggregate grading curve was not clearly observed, although slightly higher values of flexural strength were achieved by concretes with a continuous grading curve and a maximum aggregate grain size of 22 mm.

Significant differences between individual formulas, especially between mined and crushed aggregates, can be seen in the water tightness of concrete. Recipes with mined aggregates show lower resistance to the action of pressurized water, and this may directly relate to their higher absorptivity values. However, this trend was not directly confirmed by the water absorption test. The use of crushed fine aggregates in a suitable mixing ratio with natural fine aggregates seems to be highly suitable for practice based on all the achieved results. This technology of concrete production could bring a significant increase in the resistance of concrete, for example to the action of pressurized water, as evidenced by the obtained results.

**Figure 8:**
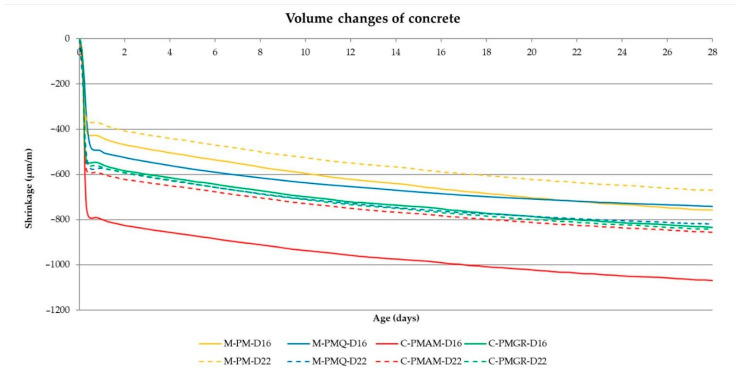
The course of the volume changes of the analyzed concrete mixtures.

**Figure 9:**
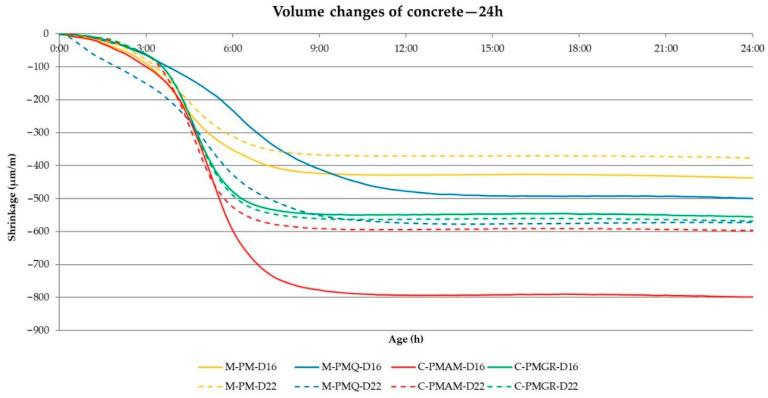
The course of the volume changes of the analyzed concrete mixtures in 24 h.

The main part of the performed experiment was to determine the impact of the type of aggregate on the volume changes of concrete. These results are shown in Table 10. In the experiment, the evaluation of the achieved volume changes after 24 h from the production of the mixtures and after 3, 7, 14 and 28 days proves to be important. Therefore, these values are included in the following Table 10 with the results. The course of the volume changes is shown in Figure 8 and Figure 9.

From the results of the volume changes of all produced concrete mixtures, it can be said that in all cases, more than 50% of the total volume changes will take place within 24 h of production (see Table 10 and Figure 8 and Figure 9).

In addition, a difference between the formulas with mined and crushed aggregates can be observed for all mixtures. Although the consistency of all mixtures was the same, the final shrinkage value is lower in mixtures with aggregates PM and PMQ, where there was also lower w/c. Based on the course of the volume changes, these results show that a major part of the volume changes is due to drying shrinkage, which corresponds to the results presented by many researchers.

**Figure 10:**
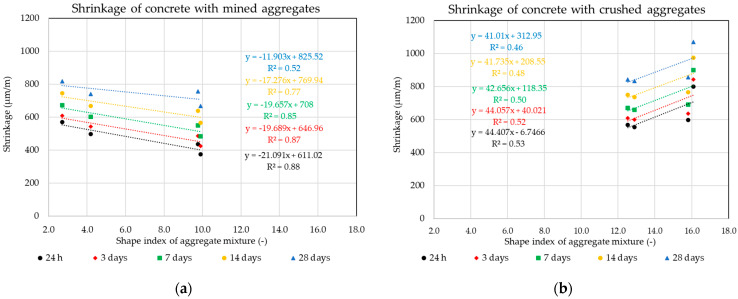
Dependence of the shape index parameters of aggregates on the volume changes of concrete: (**a**) Mined aggregate, (**b**) crushed aggregate.

**Figure 11:**
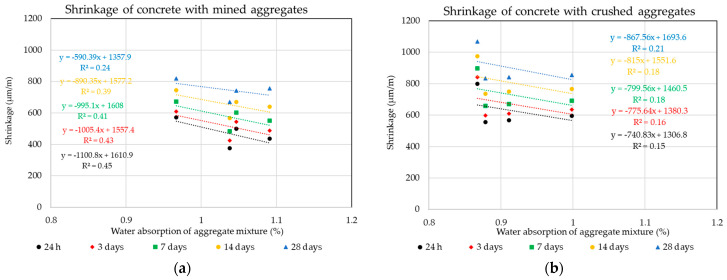
Dependence of the water absorption of aggregates on the volume changes of concrete: (**a**) Mined aggregate, (**b**) crushed aggregate.

**Figure 12:**
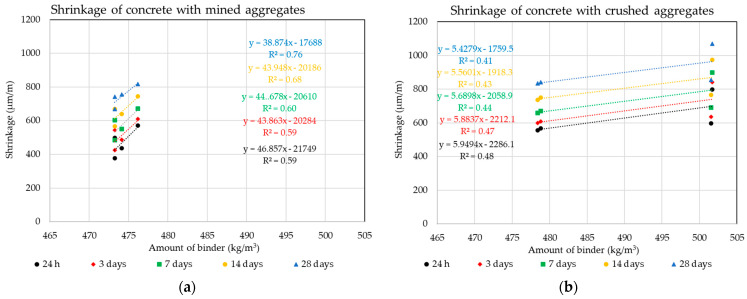
Dependence of the amount of binder on the volume changes of concrete: (**a**) Mined aggregate, (**b**) crushed aggregate.

**Figure 13:**
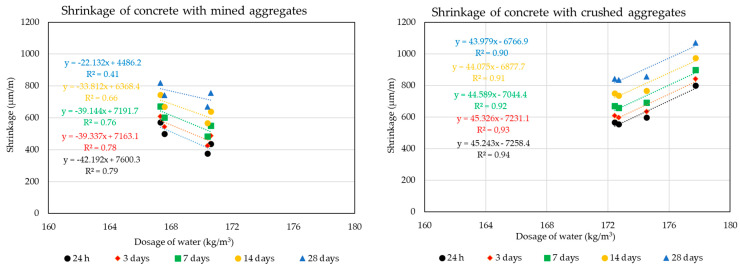
Dependence of the dosage of water on the volume changes of concrete: (**a**) Mined aggregate, (**b**) crushed aggregate.

The following graphs in Figure 10, Figure 11, Figure 12 and Figure 13 show the individual monitored dependencies, more specifically the effect of the shape index, the water absorption of the aggregate on the volume changes of concrete, the amount of particles size less the 0.125 mm and the dose of water on the volume changes of concrete.

These dependencies are further analyzed using the statistical method of Pearson’s correlation coefficient. Pearson’s correlation coefficient is a statistical indicator of the strength of a linear dependence between two quantities. It takes values from −1.0 to 1.0, and the closer the value is to 1 or −1, the stronger the linear correlation between the tested quantities. Positive values then indicate a positive linear correlation and negative, vice versa. Reaching a value of 0 means that there is no linear correlation between the monitored variables [51,52].

The water absorption values of the aggregate mixture for the individual formulas were calculated from the weighted averages according to the given composition and the representation of the individual fractions. The same procedure was used for the shape of aggregate grains using the shape index value. Due to the impossibility of including small, crushed aggregate in the shape of grains, the dependence of the amount of all fine fractions up to 0.125 mm on the volume changes of concrete was determined. The binder component and all grains of the aggregate mixture with a size of less than 0.125 mm are included in these fine fractions.

In order to more accurately determine the possible dependencies of the individual monitored parameters of the aggregate on the volume changes of concrete, the calculation of the Pearson’s correlation coefficient was performed. This coefficient was determined for a given parameter every 24 h and 7 and 28 days. The results are contained in Table 11.

The results of the statistical analysis of the results obtained using the Pearson’s coefficient demonstrates that for conventional ready-mixed concretes, the production of mixtures with a discontinuous grading curve is not unsuitable from the point of view of negatively influencing the undesired volume changes of the composite. A significant dependence was observed in the overall water absorption of the aggregate mixture on the volume changes of the composite. The results of the experiment clearly prove that although the mined aggregates used achieve higher values of water absorption, this parameter is linearly and indirectly dependent on the resulting volume changes of the composite. This fact directly relates to the linear dependence of the water dose for the production of the mixture and its resulting volume change. It is obvious that although the mined aggregates used have a higher water absorption value, lower doses of mixing water were used for the production of the mixtures with these aggregates, which had a positive effect on the resulting volume changes of concrete composites [17,18,19]. This result points to the inconsistency of published conclusions by many researchers that crushed aggregates with a larger specific surface can have a positive effect against undesirable volume changes of the composite and concrete mixtures produced using crushed aggregates, achieving lower volume changes [53,54]. The results of the experiment show that a higher dose of mixing water was required to moisten the surface of crushed aggregates, while achieving the same degree of consistency as mixtures with mined aggregates causes higher values of volume changes, especially in the first 24 h after their production. This fact is due to the higher value of shrinkage by drying, and the results show that this value is negatively affected mainly by the number of fine fractions in the aggregate and the shape of the aggregate, which indicates the specific surface of the resulting mixture of coarse aggregates.

For practice, it is beneficial to prove the effect of the type of aggregate used on the volume changes of the composite from its production to the age of 28 days. It is obvious that the effect of aggregate is most noticeable especially in the first 24 h after the production of the mixture, when the most significant volume changes occur mainly due to drying.

### 3.2. The Effect of Mineral Additives on the Volume Changes of Concrete

**Figure 14:**
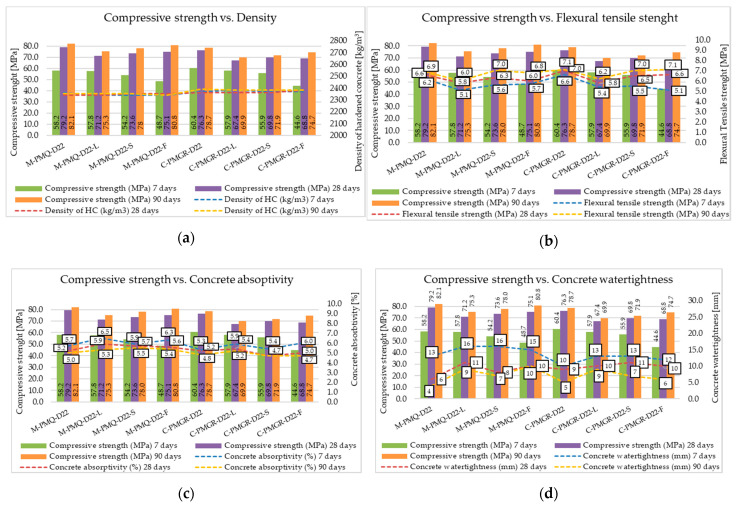
Mechanical and physical parameters of tested concretes compressive strength versus (**a**) density of hardened concrete, (**b**) flexural tensile strength, (**c**) concrete absorptivity and (**d**) concrete water tightness.

The concrete mixtures produced reached, as in the previous part of the experiment, a cone slump of about 190 mm. This degree of cone slump was again achieved thanks to a small correction of the water–cement ratio and the dose of the superplasticizer, as can be seen from the tables with the composition of individual formulas. For the mineral additives, the correction of the water–cement ratio and the dose of the superplasticizer were expected according to their grain morphology. From the analysis of the grain morphology of the mineral additives used under the scanning electron microscope, it is evident that the grains of the high-temperature fly ash are predominantly spherical in nature with a vitrified surface. For this mineral additive, a positive effect on the consistency of the concrete mixture was expected due to its addition. This fact has been proven in many research studies in which the effect of a sliding bearing is attributed to high-temperature fly ash, which have a ball shape [55]. In the performed experiment, this fact was proven despite the different amount of binder component in the individual formulas to achieve the same strength parameters. The achieved results of the fresh concrete properties are contained in Table 12, and the mechanical and physical parameters of hardened concrete are contained in Figure 14.

The required strength class C 45/55 has been achieved for all mixtures, and for the use of the active additives, their pozzolanic or latent hydraulic activity is then evident with an increasing time from production. Although the compressive strength results after 28 days show slight mutual differences, these differences do not exceed more than 13%. The results show that concretes made of quality mined aggregates, which, for this experiment, are of the quartz type, can achieve higher compressive strengths than the concretes with crushed aggregates with the same composition. Regarding the flexural strength of concrete, the results obtained are the opposite, and, in accordance with previously written conclusions, concretes with crushed aggregate achieve higher values.

**Figure 15:**
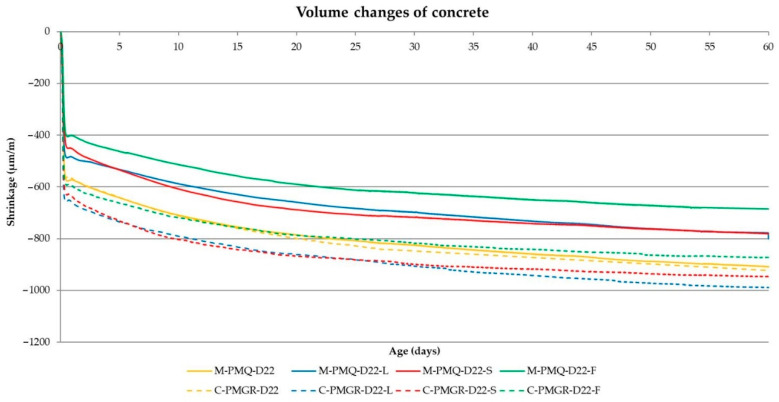
The course of the volume changes of the analyzed concrete mixtures.

**Figure 16:**
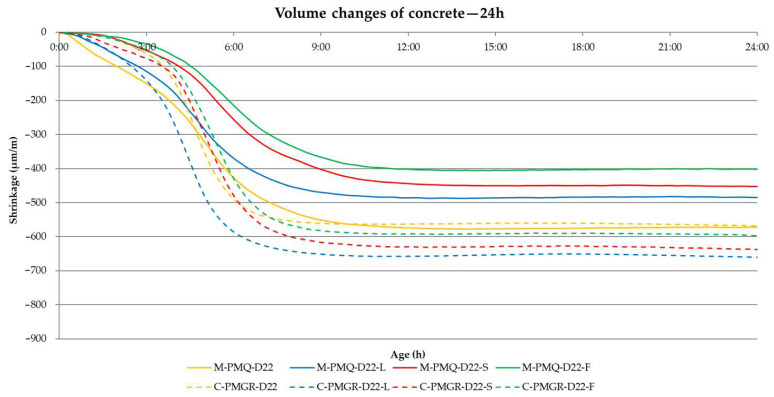
The course of the volume changes of the analyzed concrete mixtures—24 h.

The results and courses of the volume changes of the concrete mixtures are contained in Table 13, and Figure 15 and Figure 16. The performance of the test corresponds to the previous experiment described in Section 2.1.

The results of the volume changes of the individual mixtures confirm the conclusion about the most significant part of the volume changes within 24 h of production in Figure 16.

The results of the experiment indicate the suitability of using mineral additives to reduce volume changes. It has been proven that all the mineral additives used can be helpful in eliminating volume changes in concrete mixtures, where the most significant effect can be seen when using very finely ground limestone and high-temperature fly ash. For very finely ground limestone, this fact can be attributed to its practically inert nature, and for high-temperature fly ash, its grain morphology, which has a positive effect on the possible reduction of the water–cement ratio [55].

**Figure 17:**
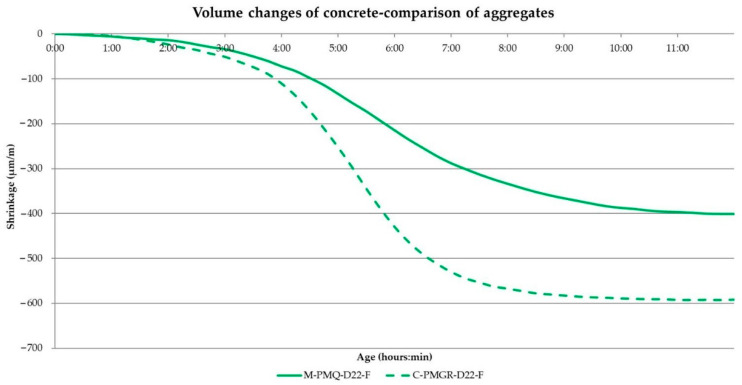
Course of the volume changes within 12 h of analyzed concrete mixtures—comparison of mined and crushed aggregates.

It also seems constructive to point out the difference in the values of the volume changes for the mixtures with mined and crushed aggregates determined by both methods. Although most of the worldwide published results describe the suitability of using crushed aggregates for volume changes of the mixture, the opposite is true when determining volume changes immediately from the production of the mixture. Based on the graphical course of the volume changes in the first hours of maturation, this fact should probably be attributed to the different loss of moisture from the mixture, when a larger dose of water to moisten the grains of the crushed aggregate causes a larger value of volume changes. This trend can be seen in Figure 16 and Figure 17, which illustratively show the course of the volume changes within 12 h of the production of a mixture of concrete and fly ash using mined, crushed aggregate.

**Figure 18:**
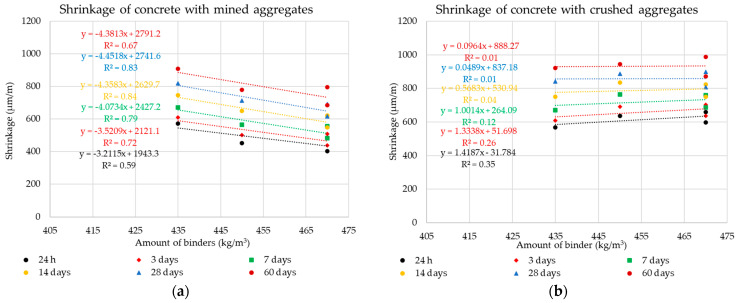
Dependencies of the dosing of mineral additives on the volume changes: (**a**) Mined aggregate, (**b**) crushed aggregate.

**Figure 19:**
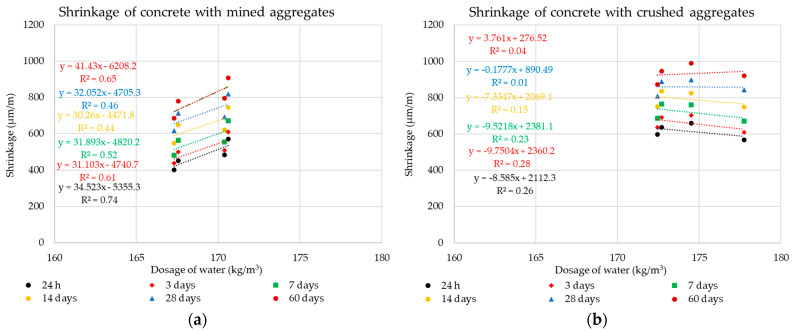
Dependencies of the dosing of water on the volume changes: (**a**) Mined aggregate, (**b**) crushed aggregate.

The following graphs in Figure 18 and Figure 19 show the effect of the dose of binder components and the amount of water on this parameter. These dependencies are further analyzed again using the statistical method of Pearson’s correlation coefficient.

The results of the calculation of the Pearson’s correlation coefficient are contained in Table 14.

Using Pearson’s correlation coefficient, it was not possible to prove a direct dependence of the amount of binder component, which is formed by Portland cement and different doses of diverse additives, on the volume changes of the composite (see Figure 18 and Figure 19). This fact testifies in particular to the different behavior of individual types of additives. It is clear from the results that even higher doses of the binder component when using finely ground limestone or high-temperature fly ash, which is increased mainly by the dose of these additives, do not have a negative effect on the resulting volume changes of the composite. The results of the experiment show that high-temperature fly ash and very finely ground limestone can be an effective way for eliminating undesirable volume changes of concrete mixtures of higher strength classes.

These results further directly relate to the dose of water required to achieve the same degree of consistency of all mixtures and the resulting volume changes. It is obvious that in this part of the study, a dependence was found between the dose of mixing water and the volume changes of the composite. This fact directly relates to the water–cement ratio, which, however, is significantly influenced by the grain morphology of the individual types of additives. The most suitable grain shape from the point of view of morphology from the images of the grains of the individual additives used (Figure 13) shows high-temperature fly ash. This shape of grain is the most suitable from the point of view of the rheology of the fresh concrete mixtures. The grains of this additive are predominantly spherical with a minimum of surface defects. The remaining two additives used underwent a grinding mechanism, which corresponds to their grain shape. For the ground blast furnace slag, a significant sharpness of individual grains is evident, which is given by their high strength and resistance to grinding. It is obvious that the process of grinding blast furnace slag has a negative impact on its effect on the rheological behavior of cement composites. For finely ground limestones, their grains are not so sharp-edged.

In addition to the effect of the individual types of additives used on the rheology of composites, their impact on the resulting volume changes of concrete is further attributed to their chemical effect. Very finely ground limestone behaves almost inertly from the point of view of chemical processes, and its use as a filler makes it possible, above all, to form a denser matrix of the composite. This can have a partial effect on the outflow of water from the composite after its production, which can have a direct effect on drying shrinkage. The course of the volume changes of the mixtures with very finely ground limestone correspond to this statement and is evident, especially with a longer maturation period. Although, for example, for the mixtures with ground blast furnace slag, shrinkage is almost complete after 28 days. For mixtures with very finely ground limestone, a more pronounced direction of the shrinkage curve can be seen even after this age. This difference between the volume changes after 28 and 60 days can still be about 15% for the mixtures with very finely ground limestone. For these mixtures, it is necessary to pay increased attention to the volume changes with a longer maturation period.

Of all the types of additives used, the use of high-temperature fly ash seems to be the most suitable for practicing eliminating negative volume changes. In addition, this additive has an incredibly positive effect on the development of hydration temperatures, which is widely used in the concreting of massive structures. In concrete mixtures, where the use of high-temperature fly ash is unsuitable, it is possible to eliminate volume changes using very finely ground limestone or ground blast furnace slag.

## 4. Conclusions

Thanks to an extensive study, the effect of the individual basic components of a concrete composite on its volume changes was clarified. The effect of the type of aggregate (mined aggregate, crushed aggregate), the characteristics of aggregate (psammite, amphibolite, quartz, granodiorite) and the aggregate grading curve (continuous and discontinuous curve) were analyzed. Furthermore, the effect of mineral additives (finely ground limestone, finely ground granulated blast furnace slag, coal high-temperature fly ash) was examined. The basic mechanical and physical parameters were determined for all the produced concrete formulas. The obtained results will allow the design of concrete composition with low-volume changes and the reduction of shrinkage cracks, which will significantly contribute to the higher durability and resistance of concrete to erosive effects and ingress of corrosion agents.

The following findings can be summarized from the results of the performed experiment:

- Maximum aggregate grain and type of grading curve—more specifically, D_max_ 16 mm and continuous grading curve and D_max_ 22 mm and continuous grading curve with omission of fraction 4–8 mm—have no significant effect on volume changes of the composite.
  ○ Mixtures with a discontinuous grading curve and a D_max_ 22 mm show slightly higher strength parameters.
- Mined aggregates are more suitable for the production of concrete mixtures with low volume changes.
  ○ Mined aggregates have a more suitable grain shape, and for the production of concrete mixtures, it is possible to use lower doses of mixing water to achieve the same degree of consistency.
  ○ A higher water absorption of mined aggregates does not have a negative effect on volume changes of the composite.
  ○ A higher specific surface area of crushed aggregate grains has a negative impact on the necessary dose of mixing water and subsequent shrinkage by drying.
  ○ The most significant effect of the type of aggregate on the volume changes of the composite is the first 24 h.
- From the point of view of the assessment of physico-mechanical properties such as compressive strength or water absorption, comparable results were obtained. The compressive strength of mined quartz-type aggregate is comparable to the compressive strength of high-quality crushed aggregate.
- In the case of the use of mineral admixtures with mined aggregate, it is clear that all used additives had a positive effect on the resulting shrinkage value of the concrete compared to the reference mixture.
  ○ The addition of high-temperature fly ash proved to be the most suitable. This fact is attributed to the reduction of the development of hydration heat by the use of high-temperature fly ash and the spherical shape of the fly ash grains, thanks to which the amount of mixing water can be eliminated.
  ○ Regarding concretes with the addition of very finely ground limestone, a more significant course of volume changes was recorded, even after 28 days from the production of concrete.
- Compared to the reference mix design with crushed aggregate, the effect of shrinkage by mineral additives is not so clear. This phenomenon is caused by the higher specific surface area of, for example, finely ground limestone, where this admixture in combination with crushed aggregate requires the use of a slightly larger amount of mixing water and plasticizer.

## Figures and Tables

**Table 1:** Parameters of used mined aggregates.

Parameter							
Petrography	Psammitic fluvial				Quartz		
Type	Mined—excavated				Mined—from water		
Locality	Zabcice (CZ)				Naklo (CZ)		
Fraction (mm)	0–4	4–8	8–16	16–22	4–8	8–16	11–22
Density (Mg/m^3^)	2.69	2.60	2.56	2.62	2.56	2.56	2.55
Water absorption (%)	1.2	1.3	0.9	0.9	1.3	0.8	0.7
Grain category (-)	GF85 *	GC85/20 *			GC85/20 *		
Fine particle content (%)	1.2	0.6	0.3	0.2	0.1	0.2	0.3
Shape index (%)	-	12	20	16	13	6	4

* Note: Grain category according EN 933-1.

**Table 2:** Parameters of used crushed aggregates.

Parameter							
Petrography	Amphibolite				Granodiorite		
Type	Crushed				Crushed		
Locality	Zelesice (CZ)				Olbramovice (CZ)		
Fraction (mm)	0–4	4–8	8–16	11–22	4–8	8–16	11–22
Density (Mg/m^3^)	2.91	2.93	2.92	2.93	2.64	2.63	2.65
Water absorption (%)	0.6	0.9	0.7	0.5	0.5	0.6	0.7
Grain category (-)	GF85 *	GC85/20 *			GC85/20 *		
Fine particle content (%)	1.4	0.3	0.6	0.4	0.4	0.6	0.6
Shape index (%)	-	28	33	29	32	23	26

* Note: Grain category according EN 933-1.

**Table 3:** Properties of used cement and superplasticizer.

Material	Parameter	
Cement CEM I 42.5 R	Compressive strength—2 days (MPa)	30.4
	Compressive strength—28 days (MPa)	60.5
	Flexural strength—2 days (MPa)	6.3
	Flexural strength—28 days (MPa)	9.1
	Grinding fineness (m^2^/kg)	414
	Specific weight (kg/m^3^)	3110
	Volume stability (mm)	0.9
Superplasticizer MC PowerFlow 2695	Density (kg/m^3^)	1070
	Color (-)	Yellow to brown
	Recommended dosage range (g/kg of cement)	2–50

**Table 4:** Composition of concrete mixtures with a continuous grading curve and D_max_ 16 mm.

Raw Material	M-PM-D16	M-PMQ-D16	C-PMAM-D16	C-PMGR-D16
CEM I 42.5 R (kg)	435	435	435	435
0–4 PM (kg)	827	820	465	868
0–4 AM (kg)	-	-	452	-
4–8 PM (kg)	231	-	-	-
4–8 Q (kg)	-	225	-	-
4–8 AM (kg)	-	-	235	-
4–8 GR (kg)	-	-	-	200
8–16 PM (kg)	700	-	-	-
8–16 Q (kg)	-	717	-	-
8–16 AM (kg)	-	-	743	-
8–16 GR (kg)	-	-	-	710
Water (kg)	168	165	175	170
water-cement ratio w/c (–)	0.386	0.379	0.402	0.391
Superplasticizer (kg)	2.61	2.57	2.74	2.70

**Table 5:** Composition of concrete mixtures with a discontinuous grading curve and D_max_ 22 mm.

Raw Material	M-PM-D22	M-PMQ-D22	C-PMAM-D22	C-PMGR-D22
CEM I 42.5 R (kg)	435	435	435	435
0–4 PM (%)	827	853	468	868
0–4 AM (%)	-	-	453	-
8–16 PM (%)	550	-	-	-
8–16 Q (%)	-	540	-	-
8–16 AM (%)	-	-	568	-
8–16 GR (%)	-	-	-	533
16–22 PM (%)	388	-	-	-
11–22 Q (%)	-	370	-	-
11–22 AM (%)	-	-	415	-
11–22 GR (%)	-	-	-	378
Water (kg)	168	165	172	170
water-cement ratio w/c (–)	0.386	0.379	0.395	0.391
Superplasticizer (kg)	2.39	2.31	2.52	2.44

**Table 6:** Quantitative X-ray fluorescence (XRF) analysis of represented oxides in mineral additives.

Mineral Additive			
Parameter	Limestone	Slag	Fly Ash
SiO_2_ (%)	0.85	32.22	58.10
Al_2_O_3_ (%)	0.62	8.07	22.20
Fe_2_O_3_ (%)	0.33	1.16	7.46
CaO (%)	56.52	48.02	3.80
MgO (%)	1.79	6.71	2.56
Na_2_O (%)	0.21	0.37	0.50
K_2_O (%)	0.08	0.79	2.72
SO_3_ (%)	0.04	0.00	0.20

**Table 7:** Properties of analyzed mineral additives.

Mineral Additive				
Parameter		Limestone	Slag	Fly Ash
Specific weight (kg/m^3^)		2710	2920	2230
Grinding fineness—specific surface—Blaine (m^2^/kg)		4210	3200	3205
Loss on ignition up to 1000 °C (%)		42.2	1.1	2.0
Efficiency index (%)	7 days	70.4	55.4	80.2
	28 days	73.4	81.4	81.4
	90 days	79.9	105.3	109.0

**Table 8:** Composition of concrete mixtures with mineral additives.

	Designation							
Raw Material	M-PMQ-D22	M-PMQ-D22-L	M-PMQ-D22-S	M-PMQ-D22-F	C-PMGR-D22	C-PMGR-D22-L	C-PMGR-D22-S	C-PMGR-D22-F
CEM I 42.5 R (kg)	435	390	360	350	435	390	360	350
Limestone (kg)	-	80	-	-	-	80	-	-
Slag (kg)	-	-	90	-	-	-	90	-
Fly ash (kg)	-	-	-	120	-	-	-	120
0–4 PM (kg)	853	808	818	798	868	808	826	796
8–16 Q (kg)	540	532	541	533	-	-	-	-
8–16 GR (kg)	-	-	-	-	535	532	545	534
11–22 Q (kg)	370	376	380	374	-	-	-	-
11–22 GR (kg)	-	-	-	-	378	385	390	384
Water (kg)	165	167	167	160	170	175	170	168
Superplasticizer (kg)	2.31	1.99	1.76	1.65	2.61	2.22	1.76	1.65

**Table 9:** Properties of fresh concrete.

	Designation of Concrete							
Parameter	M-PM-D16	M-PMQ-D16	C-PMAM-D16	C-PMGR-D16	M-PM-D22	M-PMQ-D22	C-PMAM-D22	C-PMGR-D22
Density FC * (kg/m^3^)	2350	2390	2550	2410	2350	2400	2550	2410
Consistency by slump (mm)	190	200	190	190	200	190	190	190

* Note: FC—fresh concrete.

**Table 10:** Volume changes of tested concretes.

	Designation of Concrete							
Volume Changes of Concrete (µm/m)	M-PM-D16	M-PMQ-D16	C-PMAM-D16	C-PMGR-D16	M-PM-D22	M-PMQ-D22	C-PMAM-D22	C-PMGR-D22
24 h	437	499	799	555	376	572	597	568
SD *	13.6	13.7	10.9	9.9	9.8	10.1	11.1	12.5
CV **	3.1	2.7	1.4	1.8	2.6	1.8	1.9	2.2
3 days	487	543	842	599	425	610	636	609
SD *	8.7	12.9	8.7	8.7	8.7	9.0	9.2	9.8
CV **	1.8	2.4	1.0	1.4	2.1	1.5	1.4	1.6
7 days	551	603	899	658	484	672	691	671
SD *	7.1	9.6	8.8	7.8	7.8	9.5	9.4	8.0
CV **	1.3	1.6	1.0	1.2	1.6	1.4	1.4	1.2
14 days	640	669	975	736	567	745	766	750
SD *	8.2	8.2	8.1	7.4	7.8	7.6	6.9	7.4
CV **	1.3	1.2	0.8	1.0	1.4	1.0	0.9	1.0
28 days	756	742	1070	835	669	819	856	842
SD *	6.6	5.8	7.8	4.5	7.4	6.2	5.8	5.1
CV **	0.9	0.8	0.7	0.5	1.1	0.8	0.7	0.6

Note: * SD—Standard deviation, ** CV—coefficient of variation (%).

**Table 11:** Pearson’s correlation coefficient.

Parameter		Volume Changes
Grain shape of coarse aggregate mixture	24 h	0.46
	7 days	0.47
	28 days	0.56
Water absorption of aggregate mixture	24 h	−0.76
	7 days	−0.75
	28 days	−0.75
Number of particles below 0.125 mm	24 h	0.79
	7 days	0.78
	28 days	0.79
Water dose	24 h	0.69
	7 days	0.69
	28 days	0.78

**Table 12:** Properties of fresh concrete.

	Designation							
Parameter	M-PMQ-D22	M-PMQ-D22-L	M-PMQ-D22-S	M-PMQ-D22-F	C-PMGR-D22	C-PMGR-D22-L	C-PMGR-D22-S	C-PMGR-D22-F
Density of FC * (kg)	2400	2390	2380	2380	2410	2410	2400	2410
Consistency by slump (mm)	190	200	190	200	190	200	200	190

Note: * FC—fresh concrete.

**Table 13:** Volume changes of tested concretes.

	Designation							
Volume Changes of Concrete (µm/m)	M-PMQ-D22	M-PMQ-D22-L	M-PMQ-D22-S	M-PMQ-D22-F	C-PMGR-D22	C-PMGR-D22-L	C-PMGR-D22-S	C-PMGR-D22-F
24 h	572	485	453	402	568	660	637	597
SD *	12.0	5.7	12.0	9.1	13.0	12.8	11.7	12.8
CV **	2.1	1.3	2.6	2.3	2.3	1.9	1.8	2.1
3 days	610	509	501	439	609	703	692	637
SD *	13.3	4.8	8.7	6.5	7.7	9.3	9.4	10.3
CV **	2.2	0.9	1.7	1.5	1.3	1.3	1.4	1.6
7 days	672	556	565	482	671	761	765	687
SD *	10.2	5.9	5.0	6.2	5.2	7.1	10.6	11.1
CV **	1.5	1.1	0.9	1.3	0.8	0.9	1.4	1.6
14 days	745	622	649	549	750	824	836	751
SD *	9.1	6.0	5.0	7.0	4.9	7.0	9.6	8.7
CV **	1.2	1.0	0.8	1.3	0.7	0.9	1.1	1.2
28 days	819	692	714	617	842	899	888	809
SD *	8.0	5.6	5.4	5.8	5.9	6.5	9.4	6.8
CV **	1.0	0.8	0.8	0.9	0.7	0.7	1.1	0.8
60 days	908	796	780	685	922	989	946	872
SD *	5.3	4.3	4.5	4.1	6.5	5.9	8.3	6.1
CV **	0.6	0.5	0.6	0.6	0.7	0.6	0.9	0.7

Note: * SD—Standard deviation, ** CV—coefficient of variation (%).

**Table 14:** Pearson’s correlation coefficient.

Parameter/Test		Volume Changes
Amount of binder component	24 h	−0.15
	7 days	−0.24
	28 days	−0.35
	60 days	−0.33
Water dose	24 h	0.81
	7 days	0.80
	28 days	0.85
	60 days	0.85

## Data Availability

The data presented in this study are available on request from the corresponding author.
